# Food-Based Interventions to Modify Diet Quality and Diversity to Address Multiple Micronutrient Deficiency

**DOI:** 10.3389/fpubh.2015.00277

**Published:** 2016-01-05

**Authors:** Madhavan K. Nair, Little Flower Augustine, Archana Konapur

**Affiliations:** ^1^Micronutrient Research, National Institute of Nutrition, Indian Council of Medical Research, Hyderabad, India

**Keywords:** dietary diversity, micronutrients, food synergy, diet quality, developing countries

## Abstract

Global data indicate a high prevalence of hidden hunger among population. Deficiencies of certain micronutrients such as folic acid, iodine, iron, and vitamin A have long lasting effects on growth and development and therefore have been a National priority from many decades. The strategy implemented so far limits to the use of supplemental sources or fortified foods in alleviating the burden of deficiencies. These approaches however undermine the food-based strategies involving dietary diversification as the long-term sustainable strategy. There is lack of understanding on the level of evidence needed to implement such strategies and the level of monitoring required for impact evaluation. Dietary diversity concerns how to ensure access for each individual to a quality and safe diet with adequate macro- and micronutrients. The key to success in using dietary diversity as a strategy to tackle hidden hunger is in integrating it with the principles of bioavailability, translated to efficient food synergies with due emphasis on food accessibility, affordability, and outdoor physical activity/life style modifications. Promoting enabling environment and sustainable agriculture is crucial for practicing dietary diversification with behavior change communication as an integral segment. It can be concluded that food-based strategies require careful understanding of the factors associated with it and moderate it to form an effective strategy for controlling multiple micronutrient deficiencies.

## Introduction

Globally, an estimated two billion people suffer from a chronic deficiency of micronutrients (1). In developing countries, multiple micronutrient deficiencies often occur concurrently in the same population [(2–5), Table 1]. Even mild to moderate deficiencies of micronutrients lead to impaired physical and cognitive development, poor physical growth, and work capacity and thereby considered as hidden hunger, which impact the nation’s development (6). Three-pronged strategy has been envisaged for prevention and control of hidden hunger, which can be deployed individually or in combination: short-term supplementation, medium-term food fortification, and a long-term focus on dietary diversification. Success in the past with respect to supplementation or fortification strategies could be attributed to reduction of specific clinical forms of micronutrient deficiency prevalent in the population such as Pellagra (niacin), beri-beri (vitamin B1), rickets (vitamin D), goiter (iodine), and neural tube defects (folic acid). The clinical prevalence of Bitot’s spot (vitamin A deficiency) has come down and nutritional blindness has been eliminated. However, the presence of subclinical forms of deficiency is more than 40% (7). The same situation can be attributed to anemia due to iron deficiency with severe forms of anemia reduced to <2% with mild moderate anemia now accounting to more than 50% (2). Inadequacies of vitamin D, vitamin B-12, and zinc based on biomarkers have been reported.

**Table 1:** **Global data on prevalence of major micronutrient deficiencies**.

	Anemia		Night blindness (VAD)		Inadequate zinc intake	Hypovitaminosis D
	Children <5 years	Pregnant women	Children <5 years	Pregnant women		
Africa	53	39	2.1	9.4	22	40–91
Asia	40	36	0.5	7.8	26	25–80
Europe	26	24	0.7	2.9	10	15–83
Latin America and Caribbean	33	27	0.6	4.4	17.0	27–67
Oceania	43	36	0.5	9.2	22	25–80
Global	43	38	0.9	7.8	17	30–90

*Prevalence of subclinical forms of vitamin A deficiency is 33.3% in preschool children and 15.3% among pregnant women. Severe anemia amounts to 1.1% globally among non-pregnant women and 0.9% among pregnant women. Based on Ref. (2–5)*.

Addressing mild to moderate deficiencies of multiple micronutrients evidenced by biomarkers is not easy and may not respond to single short-term intervention. This may require more comprehensive approach with package of intervention including the right cocktail of micronutrients, whole foods, and entire diets for sustaining the effects. These should be integrated in such a way that there is a smooth transition from the single- or multiple-micronutrient interventions to the most sustainable strategy of dietary diversification. The paper will focus on dietary diversification as a viable strategy for combating micronutrient malnutrition in developing countries with emphasis on challenges and opportunities and a strategic framework for diversification to work in the context.

## Advantages and Disadvantages of Dietary Diversification

**Figure 1:**
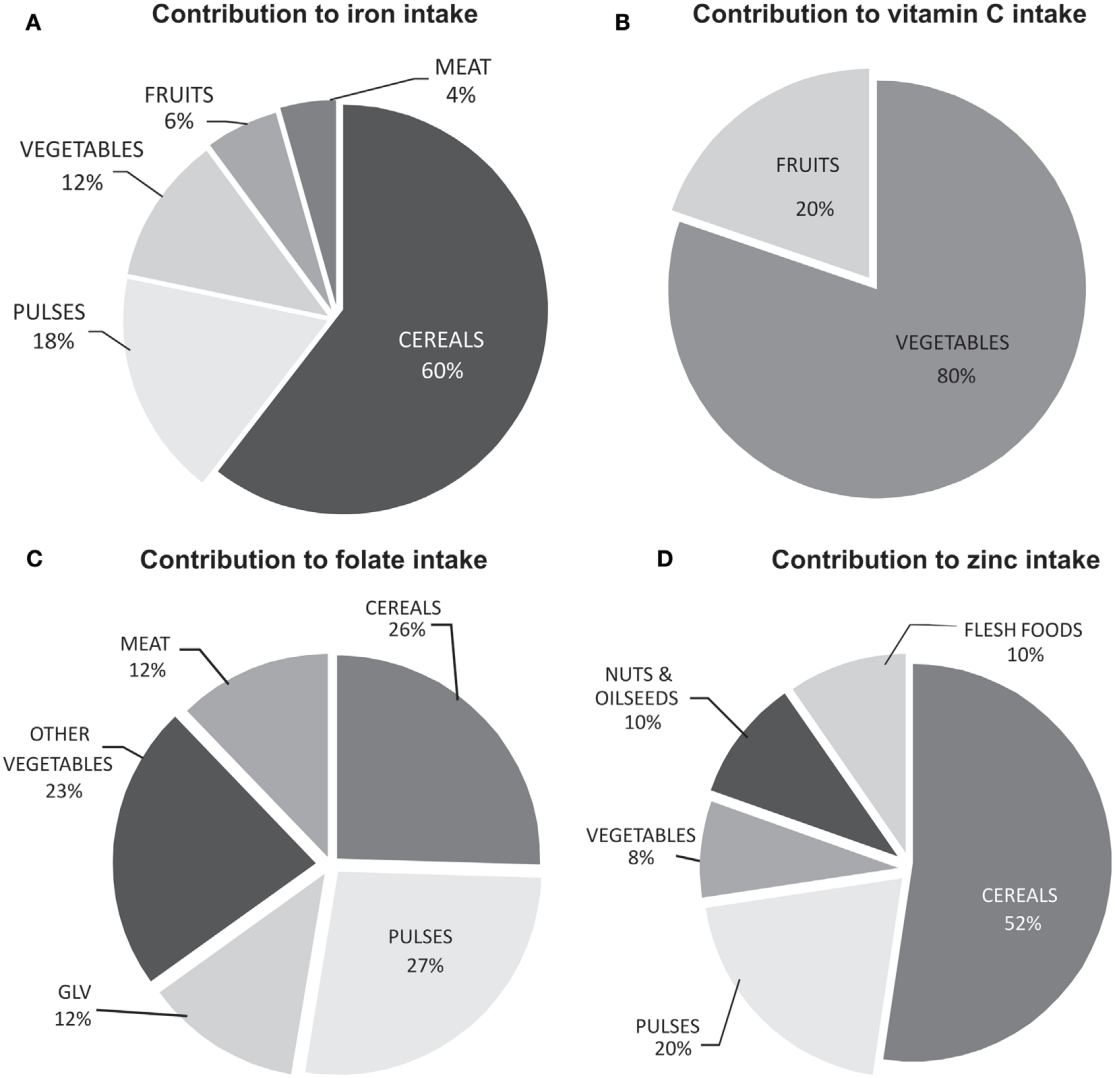
**Contribution of different food groups in meeting the daily requirement of micronutrients (A) iron, (B) vitamin C, (C) folate, and (D) zinc**. Calculated using the model diet (8) and the food composition database (9).

Acquiring all micronutrients from one or two food groups is not plausible and requires regular intake of several foods and food groups in sufficient quantity and variety to satisfy the nutritional needs. The example of four major micronutrients iron, vitamin C, folate, and zinc has been provided for reference [(8, 9), Figure 1]. For a developing country like India which is predominantly cereal pulse-based vegetarian diet with minimal amounts of flesh foods, contrary to popular assumption, cereals appear to be the major source of iron and zinc, owing to the sheer volume of intake. For vitamin C, fruits and vegetables are the sole source, and for folate, a mixture of all food groups contribute.

Strategies other than dietary diversification have the disadvantage of targeting the known factors. Addressing the “uncertainty of unknown” is an advantage for an approach aimed at diversification in the diet. Dietary diversification has an additional advantage of being more close to the population psyche and culture but also bears the challenge of breaking the inertia of habituation. Once made viable, dietary diversification is a strategy which is sustainable without external support and has the ability to simultaneously combat multiple micronutrient deficiencies (10). However, the strategy bears several disadvantages such as a lack of evidence base, slow returns, lack of measurable end points, and issues of affordability, taking the forefront which needs to be tackled during the course of implementation (Table 2).

**Table 2:** **Advantages and disadvantages of diet diversity**.

Advantages	Disadvantages
Long-term sustainable strategy	May not work as a sole strategy for vulnerable segments due to the need for long-term practice and consumption for demonstrating an impact
Emphasizes food groups (not individual foods) and food synergy	Creating enabling environment and nutrition education at all levels
Improve quality of diets and ensures improved micronutrient intake by entire household	Overestimates the benefit and ensuring adherence is challenging
Targets multiple micronutrients	Accurate food data composition is needed, difficulty in making parallelism between nutrient sources vs. biomarker
Understanding of how foods interact with the human biological system (holistic approach)	Very complex pathways and lack of measureable endpoints
Relatively low antagonistic interactions and targets unknown nutrients	Minimal processing of unconventional foods, intake of non-nutrients and antinutrients affecting health, e.g., phytate and toxins
Cultural acceptability	Lacks evidence base and employs history of safe use and practicing food fads. Requires behavioral change and knowledge of food synergy
Economic feasibility	Nutrient-rich expensive dry fruits and nuts, perishable flesh foods, fruits, and vegetables and needs cold storage
Biodiversity and employment	Unorganized sector

## Assessment of Dietary Diversity in Relation to Micronutrient Adequacy

Dietary diversity previously has been defined as the number of different foods or food groups consumed over a given reference period (11). It encompasses both inter- and intralevel variety of foods groups for which increased availability, access to and consumption of adequate quantities and appropriate varieties of safe, good quality food is a prerequisite. Dietary diversity had long been recognized as an important component of diet quality. However, the measurement of diet diversity suffered from a lack of consensus across countries with regard to the indicators and validation methods and was therefore difficult to compare. Probability of adequacy (PA) and mean probability of adequacy (MPA) are two indices which have been used to understand to what extent dietary diversity can predict micronutrient adequacy. PA is the ratio of estimated intake to recommended intake, and the MPA is the average of the PA. Nutrient adequacy ratio (NAR) and mean adequacy ratio (MAR) is another set of indicators, where MAR is the mean ratio of intake to recommended intake (each truncated at one). The major difference between PA and NAR is in terms of using the Estimated Average Intake or the recommended dietary allowance.

Guidelines have been brought out by FAO (12) with validated indices of individual and household dietary diversity for children and reproductive age women and would prove to be helpful for program planners. It is recommended to use the mean score or distribution of scores for analytical purposes and to set program targets or goals. This index has been validated for micronutrient adequacy and thereby dietary quality at individual level. At household level, it is mostly considered as indicator of food security.

Several indices such as food variety score (FVS, count of number of foods), diversity within food group, dietary serving score (DSS), dietary diversity score (DDS), healthy eating index score, and diet quality index (measure of food quality) have been tried by several researchers over the past decade among children, adolescents, adults, and the elderly. Currently, DDS is defined as the measure of number of food groups consumed over the reference period by the individual. However, the number of food groups used varies across studies and has been summarized in Table 3.

**Table 3:** **Diet diversity tools developed and validated with micronutrient adequacy in developing countries**.

Country	Target group (*n* = 70–3164)	Tools	Tool specifications, advantages/disadvantages	Correlation with MAR
Mali (13, 14)	13–58-month-old children (*n* = 77)	DDS, FVS	8 food groups for DDS and 75 food items for FVS. Simple count of foods over 24 h. Used sensitivity–specificity analysis for cutoff	Medium
	15–59-year-old men and women (*n* = 75)	DDS, FVS	9 food groups for DDS and 69 food items for FVS. Simple count of foods over 24 h	FVS medium, DDS low
	15–45-year-old men and women (*n* = 70)	DDS, FVS	9 food groups for DDS and 164 food items for FVS. Simple count of foods over 24 h	Medium
Tehran (15)	10–18-year-old adolescents (*n* = 304)	DDS	5 food groups for DDS. Quantification using half recommended serving intake during a 2-day period	Medium
Sri Lanka (16)	>60 years (*n* = 200)	FVS, DDS, DDS half DSS	6 food groups for DDS, DDS half, DSS. 15 foods for FVS. Simple count for DDS and FVS, intake of half recommended serving for DDS half, and DSS received a score of maximum of 20. Sensitivity and specificity analysis was done	Medium
S. Africa (17)	1–8-year-old children (*n* = 2200)	DDS, FVS	9 food groups for DDS and 45 food items for FVS. Simple count of foods over 24 h. Sensitivity and specificity analysis for cutoff	High
Data sets of^a^	15–49-year-old women	Food group diversity indicators	6, 9, 13, and 21 food groups. DDS quantified using minimum of 1 and 15 g intake for 24 h. Sensitivity and specificity analysis for cutoff	Low to medium for both FGI, 1 g FGI, 15 g
Burkina Faso	(*n* = 178)			
Mali	(*n* = 102)			
Mozambique	(*n* = 409)			
Bangladesh	(*n* = 412)			
Philippines (18)	(*n* = 2045)			
India^a^ (19)	5–8-year-old children (*n* = 232)	DDS	FVS	13 food groups for DDS and 78 food items for FVS. Simple count for 24 h. Sensitivity and specificity analysis for cutoff	Low
Philippines^a^ (20)	24–71 months old (*n* = 3164)	DDS	DDS 10 g	10 food groups for DDS with and without quantification (10 g minimum intake) for 24 h. Sensitivity and specificity analysis for cutoff	Medium
	24 months old (*n* = 1810)	DDS	DDS 10 g	9 food groups with and without quantification (10 g minimum intake). Sensitivity and specificity analysis for cutoff	Low to medium

*<0.1–0.29: low, 0.3–0.69: medium, and >0.7 arbitrarily considered as high correlation. A variety of indexes were developed and validated with mean adequacy ratio – MAR, which is the mean ratio of intake to recommended intake*.

*^a^Validated with mean probability adequacy percentage – MPA which is the intake to estimated average requirement. DDS, dietary diversity score; DSS, dietary serving score; FVS, food variety score; FGI, Food Group Index. These indexes include healthy eating index, diet quality index, healthy diet indicator, dietary guidelines index, Mediterranean diet score, food-based quality index, healthy food index, food pyramid index, and recommended food score*.

It has been observed that DDS based on a simple count of food groups consumed and DDS using a 10-g minimum intake for each food group (DDS – 10 g) were both significant predictors of adequate micronutrient intake in 24–71-month-old non-breast-feeding Filipino children. The best cutoff points for achieving 50 and 75% probability of adequate micronutrient intake were 5 and 6 food groups, respectively (21). However, the DDS – 10 g was found to be better indicator of nutrient adequacy among 24-month-old children from the same country (22). When DDS developed for schoolchildren in rural Kenya based on various minimum intake amounts from each food group (1 g, 15 g, a variable minimum based on the content of a target nutrient for each group, the median intake level for each group; and the 90th percentile intake level for each group) were compared, the DDS based on a 15-g minimum and DDS based on nutrient content were only significantly associated with MPA after adjusting for energy intake (23). DDS and associations with MPA or MAR has been reported from Mali among children (13) and adults (14), adolescents from Iran (15), South Africa among elderly (24), and rural elderly people in Sri Lanka (16). A DDS cutoff of 4 was found to be best indicator of MAR <50% from a secondary data analysis of a nationally represented study of 1999 among 1–8-year-old children from South Africa (17). In a recent analysis by the same group showed that all food group systems significantly correlated with MAR with 13 and 21 food groups exhibiting marginally better associations among a nationally representative sample of 1–9-year-old children (25). When analyzed, the data base on reproductive age women from Burkina Faso, Mali, Mozambique, Bangladesh, and Philippines with repeated 24 h recall data showed that the MPA and DDS are associated with each other with all types of food grouping tested for MPA calculated for thiamin, calcium, riboflavin, iron, niacin, zinc, vitamin B6, folate, vitamin B-12, vitamin A, and vitamin C. However, these studies also point toward the need for a local adaptation to arrive at the best cutoff due to variability in MPA (18). A typical example is the study conducted in Nepal among lactating women using eight food groups, wherein the MPA for 11 micronutrients was found to be at 0.19 (26), while in India, the MPA for five micronutrients among children was 0.40 (19).

## Dietary Diversity Scores and Biomarkers of Micronutrient Status

The validities reported for DDS are limited with respect to micronutrient adequacy which is a calculated parameter from 24 h recall or food frequency questionnaires (20, 27). Validating against biomarkers is essential to acquire information on the utility and applicability of DDS scale in the case of micronutrients.

Dietary diversity score had a significant positive effect on serum retinol concentration among adult women in Kenya. The score consisted of 10 food groups: cereals/tubers, meat/poultry/fish, vitamin A-rich fruits and vegetables, other vegetables, other fruit, oils and fats, dairy, eggs, pulses/nuts, and other foods (28). Increase in the number of food groups significantly increased serum ferritin and serum retinol measures among school children in Uganda (29). Though isolated attempts have been done, there is a scope for extensive evaluation in these lines since DDS is a relatively less time consuming and easy-to-use intake quantification tool compared to the traditional methods of dietary assessments.

## Intake Biomarkers for Enhanced Validity of DDS

Intake biomarker is a relatively new concept and has scope for being an adjunct to DDS if validated properly in the context. Metabolomic techniques have been used in recent years to identify biomarkers of intake (30). The advantage of “omics” markers is the use of urinary biomarkers and therefore non-invasive methodology. Another advantage is in terms of representation of food group rather than a particular food item, e.g., citrus fruits (proline betaine), fruits, and vegetables. Several urinary biomarkers have been identified for fruit and vegetable intake using controlled studies with adequate sensitivity and specificity, the utility of which need to be established. Many are still in various stages of validation (31–46). Though at present there may not be a biomarker for DDS, it is a promising area for further research (Table 4).

**Table 4:** **Diet diversity tools validated with Biomarkers of intake and micronutrient adequacy**.

	Marker	Intake data	Outcome
1	Serum (13C:12C-retinol) by GCCIRMS (31)	3 days record for fruit and vegetable	Correlation with vegetable intake
2	Plasma vitamin C (32)	Fruits and vegetables	Correlation with weighed record method
3	Urinary levels of B1, B2, B3, B6, B7, B12, folate, and vitamin C (33)	Vitamins based on dietary assessments	Correlation except for vitamin B12 over the recent 2–4 days in free-living children, young, and elderly
4	Skin carotenoid status resonance Raman spectroscopy (34)	Fruit and vegetable consumption	Positively associated with fruit/vegetable consumption (*p* = 0.02)
5	24-h urinary excretion of Ca, Mg, P, Fe, Zn, Cu, Mn, Se, and Mo on day 4 (35)	Weighed food record	Positive association for urinary Ca, Mg, P, Se, and Mo but not for Zn, Cu, and Mn
6	24-h urinary Hippuric acid by direct colorimetry (36)	3-day weighed dietary records of fruit and vegetable intake	Correlations of HA with FVJ, K intake with urinary K in children
7	Proline betaine as marker for citrus fruit consumption by NMR spectroscopic profiling (37)	Controlled study followed by validation	Sensitivity – 86.3, specificity – 90.6
8	46 putatively annotated ions, LC-MS/MS (38)	FFQ	MS/MS fragment ion were differentially abundant between the two intervention diets
9	Serum β-carotene HPLC vitamin C automated method (39)	Interview-based dietary-history method	β-carotene associated with fruit and vegetable intake and vitamin C with fruit intake
10	Urinary flavonoids as marker of fruit and vegetable intake, LC-MS (40)	3-day dietary record	Significant differences in the urinary excretion of flavonoids between subjects on diets high or low in fruits, berries, and vegetables
11	24-h and morning urinary flavonoid excretion in LC-MS (41)	Intake of fruits and vegetables	24-h urinary flavonoids increased linearly with increasing fruit and vegetable intakes
12	Fasting plasma carotenoid levels and retinol (42)	Daily fruit and vegetable servings (DFAVS)	Plasma β-cryptoxanthin, lutein, and zeaxanthin levels were positively associated with consumed DFAVS
13	Metabolomics in urine sample (43)	Coffee intake, garlic intake, fruit and vegetable intake, alcohol intake, meat intake, hypocaloric dieting	Strongest associations between fruit and vegetables intake and a glycerophospholipid and sphingolipid
14	Metabolomics in urine sample (44)	Citrus fruit Using FFQ data	Proline betaine sensitivity 80.8–92.2; specificity, 74.2–94.1%
15	Metabolomics flow electrospray–ionization mass spectrometry (45)	Intake of salmon, broccoli, raspberries, single day breakfast	TMAO and 1-methylhistidine correlated with fish intake. Increased ascorbate derivatives with broccoli or raspberries. Sulphonated caffeic acid and sulphonated methyl-epicatechin concentrations increased after consumption of raspberries
16	Non-targeted metabolite profiling of plasma samples (46)	Intake data	Glucuronidated alk(en)-ylresorcinols correlated with the intake of whole-grain products
			Plasma furan fatty acids correlated with the intake of fish

*GCCIRMS, gas chromatography coupled to isotope ratio mass spectrometry, FFQ, food frequency questionnaire; LC–MS/MS, liquid chromatography–mass spectrometry and liquid chromatography–tandem mass spectrometry; TMAO, trimethylamine oxide*.

## Dietary Diversity and Functional Outcomes

### Growth

Systematic review has shown that in economically disadvantaged countries, diet quality indices may be predictive of child growth (47). Data from 11 recent Demographic and Health Surveys (DHS) have been used to examine the association between dietary diversity and height-for-age Z-scores (HAZ) for 6–23 month-old children, while controlling for household wealth/welfare and several other potentially confounding factors. Dietary diversity was significantly associated with HAZ, either as a main effect or in an interaction in ten out of eleven countries (48). Evidence links dietary diversity with child stunting among 11-, 12–23-, and 24–59-month-old children of rural Bangladesh (49) and in a nationally representative sample of 6209 children aged 12–59 months in Cambodia (50). Dietary diversity has been associated with anthropometry in 6–23 month-old children of Burkina Faso also (51). Stunting has been proven to be resistant to major nutritional interventions, and therefore this association provides more light into the need for holistic approaches in targeting any nutritional issue. One prospective study looked at consumption of different foods and food groups by mother during different periods of gestation, on birth weight of the offspring. Interestingly, energy and protein intakes of mothers did not show any association with the birth size of the child but fruit, vegetable, and milk consumption did show an association (52).

### Cognition

Apart from stunting, dietary quality if not diversity alone has been linked with functional outcomes such as cognition in the elderly (53–55). An association between Mediterranean diet and lesser cognitive decline has been reported by several researchers in the case of elderly (56). Cognitive performance has also been associated with dietary diversity among other children (57).

### Gut Health

Diet provides nutrients not only for the host but also for the bacteria therein and therefore is one of the major determinants of gut micro biome (58). Diet *per se* has been unequivocally linked to gut micro biota by several studies (59–62). Fruit and vegetable intake has also been recognized as capable of altering gut micro biota (63).

A linkage between dietary diversity and microbial diversity in the gut is a relatively new concept. Using fish as the model, such an association has been elucidated, i.e., more diversity of food by the fish, less is the diversity of microorganisms in the gut which is an undesirable effect. This study done in vertebrate species if confirmed in humans suggest that multiple diet components can interact non-additively to influence gut microbial diversity and point toward intelligent food synergies for a diverse gut micro biome (64).

### Preeclampsia

Dietary diversity has been linked to less reporting of a major pregnancy related complication, i.e., preeclampsia and eclampsia (65).

## Co-Benefits of Dietary Diversification

Food-based dietary diversity strategy has social, cultural, economic, and environmental benefits. Agricultural diversity not only improves production but also includes income generation and improves accessibility. Biodiverse crops will ensure sustainable diets that are environment healthy, strengthen local food systems by producing traditional/indigenous crops, and provide fodder for the livestock. Home gardening, livestock production, aquaculture, and nutrition education will empower women to improve diet quality and nutritional status of the family. Increasing demand for diversified diet with improved postharvest processing infrastructure will improve food market trade at international level.

## Successful Dietary Diversification Interventions in Developing Countries

The homestead food production by Helen Keller International significantly improved dietary diversification in Bangladesh, Cambodia, Nepal, and Philippines through home gardening, animal husbandry, and nutrition education. Animal food consumption improved among program households, with liver consumption increasing from 24% at baseline to 46% at end line and the median number of eggs consumed by families per week increasing from 2 to 5. The number of varieties of vegetable produced and the volume produced from home gardens was higher. The diversity of vegetable consumption by young children increased from four types of vegetables to 13 types of vegetables. Frequency of consumption of vegetables by children was also 1.6 times higher. The sale of such products also improved household income and the families used this income to purchase additional food for the household. Anemia prevalence among children in program households decreased in all the countries (66).

Impact of women’s empowerment in agriculture and production diversity on dietary diversity and anthropometric outcomes of mothers and children were investigated in Nepal. Production diversity was positively associated with mothers’ dietary diversity and body mass index. Production diversity was positively associated with dietary diversity for children under two and predicts weight-for-age (WAZ), weight-for-height (WHZ) *z*-scores, and HAZ of children over 2 years of age. Indicators of empowerment were significantly associated with maternal outcomes but have a variable effect on child outcomes. Women’s autonomy in production and hours worked improved maternal and children’s dietary diversity and child HAZ (67).

A study in India demonstrated the effectiveness of a social-marketing strategy for nutrition communication by promoting consumption of vitamin A-rich foods. Knowledge, attitude, and practices about vitamin A improved significantly among mothers of preschool children and a fourfold increase in the intake of vitamin A-rich foods among preschool children in the experimental area after intervention. A significant decrease in the prevalence of Bitot’s spots after intervention in the experimental area (*p* < 0.05) was observed (68).

Though only partially explored, these experiences provide evidence that dietary diversification strategy is a feasible option, provided that it is strategized and tailored to the population that it is catering to, and can be the most sustainable strategy effecting micronutrient nutrition across developing countries.

## Dietary Diversification: From Evidence to Implementation

It is imperative to understand the challenges and opportunities for promoting dietary diversification as a sustainable strategy. Though the identified challenges and opportunities may show geographical variations, the basic framework can be applied throughout and has been presented in Box 1. The challenges may spread across, from lab to the land and from there to changing the attitude of the population by and large with progressive infiltration and a positive coalition of multiple stakeholders. This full cycle of intervention has to be a slow but rewarding process. A detailed recount of how such a concept would work in developing countries has been provided in the following sections.

Box 1 **Challenges and opportunities for dietary diversification in developing countries.**

**Table:** 

Challenges	Opportunities
Policy	Evaluating the effects of government policies for production and trade on supply and demand for certain types of food and the impacts on nutrition
Creating evidence base to improve the diet quality (e.g., by increasing fruit, vegetable, and animal product intake)	How to establish a dynamic national food composition data base, recommended dietary allowances, and dietary guidelines? Can it be harmonized?
	What are the effective food synergies that can be practiced to improve micronutrient intake and bioavailability?
	Assessing bioavailability of micronutrients from modified habitual diets in vulnerable segments of the population (pregnant, lactating, children, and adolescents)
	Developing tools to assess effects on functional outcomes such as growth and development, gut health, immune function, pregnancy outcome, life style disease
	Developing biomarkers of intake and food-based systems biologyEstablishing need for complementary interventions to ensure adequate intake
Creating enabled environment	How to increase the accessibility and availability of foods produced at national level as well as practicing homestead food production at household level
	Technology of reducing postharvest losses to ensure increased shelf life
	How to engage the village leaders to improving the access of fruits and vegetables to local markets
Affordability: by providing minimum support price and cash transfer to the farmers	Assessing the economic viability and environmental sustainability
Behavior change communication for practicing diet diversification	Popularizing effective food synergies, assessing how agricultural diversity affects dietary quality in different contexts
Women’s economic empowerment and skill development for homestead production	Assessing enhanced micronutrient status for women and children
Developing nutrition-centered approaches in climate change	Understanding the relationship between factors (food system and nutrition, individual- and household level-factors, and climate change/population trend) and outcomes (good nutrition, economic viability, and environmental sustainability) through modeling
Post-harvest technology	Developing technologies to increase shelf life to retain all the micronutrients

### Evidence and Policy

Engaging policy makers and other stakeholders such as farmers, villagers, and technologists for creating evidence based policy is a major prerequisite for dietary diversification strategy to materialize. The prioritized policy decisions would spearhead the major change which needs to be ingrained in the population.

Existence of a food composition database with key summary tables is primary to the success of dietary diversification. Availability of these databases in the public domain is also important and is vital for the target group and the message carriers for effective health promotion. Effective dietary guidelines, food guide pyramids and nutrient requirements or recommended dietary allowances brought out by each Nation would form the concrete base upon which the diversification strategies would be built.

National guidelines do exist on how dietary diversity can be achieved for better overall health and can form a guideline at population level. The food pyramids constructed by Nations are ethnicity-specific and thereby appear to be contextual and culturally acceptable. However, the dimension of proportionality is not entirely addressed in food pyramids of several countries and need further imploration.

#### Food Synergy as an Integral Intervention Strategy to Improve Bioavailability of Micronutrients

Apart from the overall dimension of dietary diversity, utilizing the effects of favorable food combinations, need to be sufficiently elucidated for food-based approaches to succeed. But it need to be borne in mind that the micronutrient status *per se* may have an important role in regulating bioavailability which is proven at least in the case of iron (69).

Creating evidence and popularizing intelligent food synergies are required for overcoming the dueling effects of malnutrition. Food synergies are the additive or more than additive influences of foods and food constituents on health (70). Though not extensive, it is fortunate that there have been attempts both at lab and land to discern the nature of this synergy in the case of at least some of the micronutrients (71–88). However, it is a consumer-centric strategy which requires effective dissemination among the target group especially the care-givers who are often the change agents of the family with respect to nutrition.

##### Food Synergy for Iron Bioavailability

A typical example is iron which is an exceptional mineral and has the major homeostatic control at the site of absorption. Absorption of iron and zinc from vegetarian Indian meals is low (89, 90). Studies both *in vitro* and *in vivo* have demonstrated a synergistic effect of vitamin C, both in synthetic and food form on iron owing to its reducing property.

The molar ratios of ascorbic acid (AA) to iron for iron absorption have also been studied. Stable isotope studies have reported iron molar ratios of 2:1 or 4:1 increased iron absorption by 270 or 343%, respectively, in control subjects and by 291 or 350%, respectively, in subjects with iron deficiency anemia (91). Using radio-isotopic studies, Ballot et al. (71) have shown that papaya and guava having the maximum iron enhancing property. Stable isotope studies done in adolescents have also shown that inclusion of 100 g of guava in habitual meal had iron enhancing property at an AA molar ratio of around five (73). Therefore, fruits and minimally processed vegetables high in AA can enhance bioavailability of iron, if included in habitual meals.

Meat and oily fish are also apparently good promoters of iron absorption. However, when these two were compared in one study, meat appeared to be a better promoter than oily fish. This has been shown by a randomized cross-over trial of two 8-week periods in 25 young women. Though there were no significant differences across two groups, ferritin moderately decreased and sTfR increased with oily fish while the opposite happened in red meat group (79). Speculations have been made regarding cysteine-containing peptides being the meat factor of muscle proteins enhancing iron absorption. However, it has been demonstrated that cooking meat at 70, 95, or 120°C increased non-heme iron absorption by 0.9, 0.7, and 2% and is an argument against sulfydryl groups from cysteine residues in promoting iron absorption and the content decreases with increasing temperature (76). Lactic fermentation of vegetables is another interesting prospect, which has been reported to increase hydrated Fe^3+^ species and enhance iron absorption with low and high phytate meal (92).

##### Food Synergy for Provitamin A Bioavailability

Absorption of provitamin A is yet another example which has been explored with respect to fat, or other food components. This is especially important where provitamin A carotenoids contribute predominantly to vitamin A in the diet and have low efficiency of conversion (8). The amount of fat required is however inconclusive with reports of quantities as low as 2.4 g/meal possessing the promoter effect.

Apart from the presence of fat, food matrix effects such as processing may also influence the bioavailability of carotenoids. For instance, enzymatic disruption of cell wall structure enhanced bioavailability of beta-carotene from whole and minced spinach (93). Competitive inhibitory interactions have been found among the different carotenoids from various vegetable sources but did not diminish plasma carotenoid concentration (94). Surprisingly, consumption of daily recommended vegetables in one meal rather than smaller doses over multiple meals with oil exhibited the greatest absorption of carotenoids (95). Amount and source of lipid may also affect carotenoid bioavailability. Mono unsaturated fatty acids rich canola oil trended toward a higher alpha carotene and lutein absorption compared to saturated fatty acid rich butter (96). Comparison of a saturated fat source (coconut oil rich in medium chain triglycerides) with a polyunsaturated fat source (safflower oil) on carotenoid bioaccumulation from tomato has been carried out in Mongolian Gerbril. It was observed that coconut oil enhanced tissue uptake of tomato carotenoids to a greater extent than safflower oil. Coconut oil increased serum cholesterol but decreased hepatic cholesterol compared to safflower oil (97). Apart from the examples provided, an extensive recount of the carotenoid re-absorption from plant sources in the presence or absence of dietary fat has been provided elsewhere (98). However, fat intake *per se* has been reported to be meager in rural populations of India (~15%) and is also a concern how judiciously it can be included in even in the diets of urban population (99).

##### Food Synergy and Zinc Bioavailability

In the case of zinc, the protein quality and quantity, phytate and fiber, calcium, iron, cadmium, copper, and several low molecular weight ligands have been implicated and reviewed elsewhere (100). However, with the available evidence including even a small amount of animal protein from fish, poultry, guinea fowl, rabbit, goat, or eggs increases zinc (as well as non-heme iron) absorption. This enhancing effect has been linked with certain amino acid and cysteine-containing peptides released during the digestion of animal protein, forming soluble ligands with zinc. Phytate reducing properties such as soaking, germination and fermentation can also be utilized for improving zinc bioavailability at household level (101).

##### Vitamin D, Calcium, and Physical Activity

Tropical countries have an advantage of ample sunshine which needs to be harnessed for attaining vitamin D sufficiency and calcium balance. While life style modification with more emphasis on outdoor physical activity may prove to be beneficial for one segment of population, more evidence need to be brought out regarding strategies to be adopted in situations where sun exposure does not seem to be the sole reason. Built environment conducive for safe sun exposure may also prove to be beneficial (102).

#### Food Antagonisms and the Role of Food Processing

The existence of food antagonisms have been demonstrated in the presence of phytates, tannins, and oxalates with respect to iron, zinc, and calcium (103). Appropriate processing of food can bring about significant improvement in micronutrient bioavailability in terms of increase the physicochemical accessibility of micronutrients, decrease the content of antinutrients, such as phytate, or increase the content of compounds that improve bioavailability. The common methods include thermal processing, mechanical processing, soaking, fermentation, and germination/malting (104, 105). One systematic review has shown some benefit of cooking in iron pots on iron absorption. But knowing the limitations of iron absorption in man, it is important to understand the mechanism clearly before concluding on this strategy (106). Tea in a rice-based meal has shown inhibitory effects on iron absorption. The consumption of 1 or 2 cups of tea decreased iron absorption in the non-anemia subjects by 49 or 66%, respectively, and in the iron deficiency anemia group by 59 or 67%, respectively (91) and is yet another example for the need of intelligent food synergies. Inhibitory effect of dietary soluble fibers such as pectin, guar, alginate (mean decrease 33–43%) on beta-carotene have also been demonstrated (107).

### Changing Attitudes and Addressing Affordability and Acceptability: The Role of Behavior Change Communication

Approaches for promoting diversified diet brings us back to the loop of affordability and acceptability and brings out a major question on whether overall growth in economy should be the center point for advancement in health and nutrition. A case study from India would provide some clue. The time trends in expenditure of Indian population mapped by the National sample survey organization shows that expenditure on non-food items are on a rise while the money spend on food is decreasing over the past decade (108). Similarly, the pattern of expenditure on food shows an expenditure on processed food almost at par to that of staples. Thus, the issue for developing country may not just be affordability, but also the changing attitude toward consumer goods other than food. How to put back the emphasis on food brings out the importance of communication for behavior change, which is the only channel for sustenance of any nutrition programs.

Lack of involvement of the target population can kill an innovative intervention strategy. In India and several other fast developing countries, the major lacuna in building and rolling-out of a nutrition intervention program is the scarcity of behavior change communication. Health promotion though a tedious and time consuming ensures sustainability to any population based programs and therefore is worth investment. It has been identified previously that this investment is meager in India and require advocacy among stakeholders (109). Behavior change communication should revolve around the family with caregiver as the central point and decision maker of the family as an important target. However, this cannot be the concern of a single sector but requires coalition of cross-sectoral multi-stakeholders for effective implementation. Lack of validated tools for measurement of behavior change may also hinder the process of building good health promotion programs. Of late there has been an increased awareness on the issue of psychometric validation of the instruments used for behavior change (110, 111). Need for targeted nutrition education messages which are relatively easily understood and ready to implement is yet another important issue to be focused. For instance, in a predominantly cereal-pulse based vegetarian diet, enhancing micronutrient content *per se* may not be of desired benefit, but focusing on bioavailability would be. Therefore, messages of this nature need to be tested for its acceptance and popularized for effective action.

### Integration of Agriculture and Nutrition to Address Affordability

Policy decisions from Government that will create an enabling environment for food-based approaches in terms of production, storage, and safe distribution, and making it available at affordable price is required. Experiences in the past indicate that integration of smallholders into national and global food systems, emphasis on agriculture as an engine of growth and poverty reduction, national economic transformations with policy support for agriculture have reduced poverty and in turn, supported improved nutrition, especially in rural areas (112). The known strategies of homestead production of vegetables, fruits, fish, and poultry, prevention of postharvest losses, and soil health are a few strategies which need to be provided sufficient importance before we proceed toward an effective food-based strategy.

Though agriculture–nutrition integration may be of immense value in a predominantly agriculture-based Nation like India, the ownership of land becomes a prerequisite and thereby limits the benefits of this strategy to the fragment of population who own land. This brings out a major question, how the benefit of food-based approaches can percolate to the urban and landless poor and would be the major workforce in urban and rural settings? Answering this question is tricky, as the food-based strategies are based on bottom-up initiation approach. One documented strategy is a modified homestead food production model which aims at skill building and income earning opportunities linked to agricultural production and homestead production using technologies that maximize yield and could be produced in micro pots (113). Experiences from the Ethiopian Productive Safety Net Program (PSNP) comparing food supply versus conditional cash transfer has shown that the households receiving cash had better household DDSs and higher consumption of oils and fats and vitamin A-rich foods than households receiving food (114). Conditional cash transfers have exhibited increased food consumption benefits in countries such as Colombia, Mexico, and Nicaragua (115) and also with respect to overall health (116). Anticipation of overall economic growth to percolate to this segment of population will take longer time in exhibiting any discernible effects with respect to nutrition, and therefore this strategy may be of intermittent value provided that the cash transfers are index linked to food price fluctuations.

### Monitoring and Evaluation Issues

Monitoring and evaluation of changes in food pattern, food systems and food environment as a function of change in economy would help us keep track of the drastic as well as gradual shifts occurring in the population. Comprehensive national nutrition surveys are powerful tools for monitoring the nutrition scenario. In Asia, 15 countries have national nutrition surveys with a regularity of 3–5 years and Thailand, Vietnam, and Pakistan with 10 years (117). In addition to dietary intake, the surveys need to capture information on clinical and subclinical deficiency of micronutrients in order to analyze the nutrition scenario comprehensively.

Monitoring and evaluation becomes important due to the considerably long duration required for any food-based strategy to show impact. Food fortification is a typical example. The risk–benefit analysis compelled the Nations to introduce food fortification even in the absence of robust evidence structure only to terminate it in the long run when they come across a potentially harmful or a negligible effect. There had been experiences in the past, with respect to folic acid in the West, the program intended to target neural tube defects through food fortification. However, that for each neural tube defect prevented, several hundred thousand people were exposed, without choice, to extra folic acid but considered to be of no harm based on the judgment of “more-is-good” (118). After decades of folic acid fortification, evidence has emerged in terms of certain harmful effects of such an initiative in aggravating vitamin B-12 deficiency and evidence for an inverse U-shaped relation between total folate intake and Natural Killer like cell (N-K cell) cytotoxicity. N-K cells are an important part of the non-specific immune response and can kill tumor cells and virally infected cells (119). It has also been observed that those with a low vitamin B-12 status (serum cobalamin < 148 pmol/L) and high serum folate (>59 nmol/L) had an odds ratio for cognitive impairment of 5 compared with those whose vitamin B-12 status and folate status were both normal (120). This persuaded the scientific community to rethink on the principle of the “history of safe use” which is often applied to minerals and vitamins. An effective monitoring platform would provide evidence on any such drastic shift especially when several strategies are used simultaneously to address micronutrient malnutrition.

### Role of Traditional Food Systems

Exploring the traditional food systems for the protective effects offered in the past and transmitting these effects to the future need to be thought about. Though not in the case of micronutrients, the concept has been a matter of focus in the case of non-communicable diseases. The Mediterranean diet patterns originating from countries surrounding the Mediterranean Sea including France, Italy, Spain, Morocco, and Greece, a part of the world which exhibited low prevalence of non-communicable diseases exemplifies the need to explore traditional food systems. However, it needs to be borne in mind that the reproducibility of the benefits in such situation depends on a complex set of variables including geo-position, environment, ethnicity, and culture (121). The traditional food systems in India have not been researched in its full potential though methodology for documenting traditional food systems of indigenous populations have been brought out by International agencies and several indigenous populations have been studied (122). Owing to the transition which has already set in, the ways and means to research this issue need to be explored for the effective preservation of traditional food systems.

## Pathway of Achieving Sustainable Strategy of Dietary Diversification to Improve Micronutrient Status

**Figure 2:**
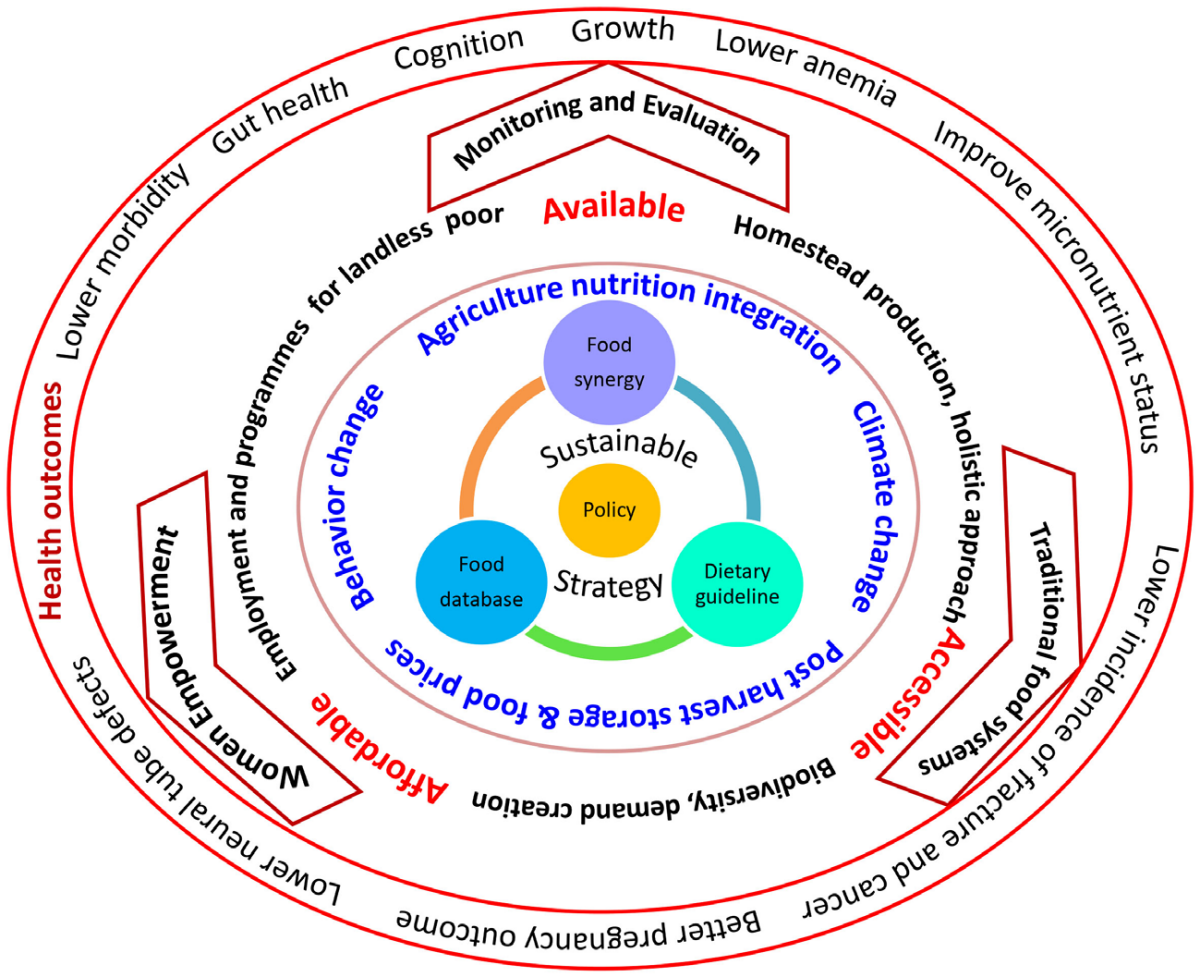
**Path of achieving sustainable strategy of dietary diversification to improve micronutrient status: a conceptual frame work for developing countries**.

A conceptual frame work for achieving sustainable strategy of dietary diversification to improve micronutrient status for developing countries is given in Figure 2. Modifications in prioritizing policy decisions are the need of the hour. Policies should work on making the food environment enabled at community level by improving access, availability of different foods and affordability of the community. For adopting sustainable strategy there is a need to generate evidence through food database and dietary guidelines are complementary to create food synergy. A proper agriculture policy to diversify food crops with post harvest processing and storage technologies can stabilize the food prices. Major efforts needs to be mandated for behavior change in the community to practice culturally acceptable affordable food synergies. Providing financial support to farmers at national level to diversify the agriculture; to individuals at household level to practice homestead food production will not only improve the availability of variety of foods but also will be a source for women empowerment and income generation which would be holistic approach. An emerging concern is the climate change which essentially can be addressed through global efforts impacting the national and local agricultural practice. In addition, establishing postharvest storage facilitates at community level will also aid in sustained quality food availability during different seasons. Foods can be made affordable by controlling food prices by facilitating trade and distribution through increased access and reduced retail prices. Women’s empowerment and community participation needs to be connected to better nutrition status through behavior change communication by encouraging them to consume foods grown at home and to utilize the money to purchase more nutritious food earned through homestead food production. A continuous monitoring and evaluation is essential to establish the health benefits and sustainability of the strategy.

## Conclusion

Promoting food-based strategies is in the nascent stages of development in developing countries. Solution-oriented transdisciplinary research with effective innovation and implementation involving multiple stakeholders and effective public–private partnership is required to tackle the problem of micronutrient insufficiency using food-based approaches. Assuming economic transition to be a solution to all problems may not be effective and therefore targeted strategies are required. Food systems that would allow healthy choice of food and facilitating the process of intelligent food synergies with home as the center point of the activity and caregiver as the change agent with effective participation from family members is required. Tracing the path that leads these changes and intervening at these critical points may result in overall health and nutrition security especially micronutrient security. Built environment changes for effective physical activity modifications and strengthening behavior change communication is required for food-based strategies to succeed.

## Author Contributions

KN was responsible for the concept, sequenced manuscript, and critically revised the manuscript. LA carried out the literature survey, compiled, and wrote first draft. KA was responsible for the data presented in tables and figures. All authors contributed to critical revision of the manuscript.

## Conflict of Interest Statement

The authors declare that the research was conducted in the absence of any commercial or financial relationships that could be construed as a potential conflict of interest.
